# Usability of a Patch-Type Ultrasound System for Non-Invasive Hemodynamic Monitoring: A Simulation Study in Anesthesiologists

**DOI:** 10.3390/healthcare14070971

**Published:** 2026-04-07

**Authors:** Soyeon Noh, Hyungmin Kim, Hyeonkyeong Choi, Wonseuk Jang

**Affiliations:** 1 Department of Medical Device Engineering and Management, Yonsei University College of Medicine, Seoul 06229, Republic of Korea; nsy4094@naver.com (S.N.); mahoyo-_0412@naver.com (H.K.); hyeonkyeong97@daum.net (H.C.); 2 Medical Device Usability Research Center, Gangnam Severance Hospital, Yonsei University College of Medicine, Seoul 06230, Republic of Korea

**Keywords:** hemodynamic monitoring, patch-type ultrasound, usability evaluation, human factors engineering, non-invasive

## Abstract

**Background/Objectives**: Non-invasive hemodynamic monitoring technologies are being developed to support clinical decisions while reducing risks from invasive procedures. Usability evaluation is essential to assess safety and effectiveness before commercial release. This study examined the usability of a novel patch-type ultrasound-based system (CW10) designed for continuous monitoring in perioperative settings. **Methods**: A summative evaluation was conducted following IEC 62366-1 with 15 anesthesiologists. Potential hazards were identified via the FDA MAUDE database (Code: DQK) to inform test scenarios. Participants were stratified by clinical experience (1–<5, 5–<10, and ≥10 years) to observe potential variations in operation. In a simulated operating room, users performed 9 clinical scenarios (49 tasks). Metrics included task success rates, subjective satisfaction (5-point Likert scale), and the System Usability Scale (SUS). **Results**: The overall task success rate was 98.2%. No statistically significant differences were observed across groups in performance, subjective ratings, or SUS scores (*p* > 0.05). The mean SUS score was 78.5, corresponding to a “Good” usability level. While some use errors occurred in tasks like probe orientation, root cause analysis suggested these were likely due to negative transfer from prior device experience rather than interface complexity. **Conclusions**: The results suggest the system demonstrates acceptable usability and consistent operation across experience levels. Integrated automated features and the patch design may contribute to reducing inter-user variability for continuous monitoring. This study provides usability evidence that may inform the development of similar non-invasive technologies.

## 1. Introduction

### 1.1. Hemodynamic Monitoring System

Hemodynamic monitoring is a critical component in the management of critically ill patients and those undergoing high-risk surgical procedures, as it is directly associated with patient prognosis. The primary objective of hemodynamic management is to maintain adequate oxygen delivery that meets tissue metabolic demands, thereby preventing cellular hypoxia and subsequent organ dysfunction [1,2]. In particular, cardiac output (CO) is a key parameter reflecting systemic blood flow and serves as an essential reference in the management of patients with shock and during the perioperative period [2,3]. Although static parameters such as central venous pressure (CVP) were traditionally used, recent clinical practice has increasingly emphasized goal-directed therapy based on dynamic indices that more accurately predict fluid responsiveness [1,3,4]. Appropriate hemodynamic monitoring plays a pivotal role in preventing complications associated with both fluid overload, such as pulmonary edema, and hypovolemia-induced impairment of tissue perfusion [5,6].

Advancements in hemodynamic monitoring technologies have focused on reducing invasiveness while preserving measurement accuracy, and current modalities are commonly classified as invasive, minimally invasive, or non-invasive. Invasive monitoring techniques, including pulmonary artery catheter (PAC), have long been regarded as the clinical gold standard for cardiac output measurement [7,8,9]. However, the use of intravascular catheters is associated with risks such as infection, arrhythmia, and pulmonary artery rupture, and reports indicating limited survival benefits have contributed to a decline in their routine use [2,7,10]. Transpulmonary thermodilution was introduced as an alternative approach; nevertheless, its application is restricted by the intermittent nature of the measurements [1,11].

Minimally invasive monitoring methods provide hemodynamic information with reduced invasiveness compared to conventional invasive techniques; however, several studies have reported limitations in measurement accuracy. For example, uncalibrated pulse wave analysis estimates vascular characteristics based on patient demographic data and algorithm-derived assumptions without external calibration [12]. This approach may fail to accurately reflect true cardiac output under rapidly changing hemodynamic conditions [13,14]. Techniques involving esophageal probe insertion include esophageal Doppler and transesophageal echocardiography (TEE). Esophageal Doppler measures blood flow velocity in the descending aorta using a flexible ultrasound probe placed in the esophagus [5,15], whereas TEE additionally allows direct visualization of cardiac structures, contractility, and valvular function [16]. However, these methods require esophageal probe placement and patient sedation, limiting their applicability in awake patients [5,17,18].

Non-invasive monitoring techniques enable cardiac output assessment without skin incision or instrument insertion, thereby enhancing patient safety. Transthoracic echocardiography (TTE) plays a central role in identifying the etiology of shock and evaluating treatment response by visualizing cardiac structure and function. Nonetheless, TTE relies on manual probe manipulation by the operator and is therefore primarily used for intermittent assessments rather than continuous monitoring. Moreover, image quality and interpretation are highly dependent on operator expertise [19]. As an alternative for continuous monitoring, the volume clamp method using a finger cuff has been employed; however, signal acquisition and accuracy may be compromised in patients with severe peripheral vasoconstriction or edema [20]. Thoracic bioimpedance and bioreactance techniques offer the advantage of simple electrode attachment and non-invasiveness, but their accuracy remains controversial due to susceptibility to electrical noise, patient motion, and pulmonary edema [5,7].

Recent advancements in non-invasive hemodynamic monitoring technologies have focused on improving measurement accuracy and enabling continuous real-time assessment through the integration of advanced signal processing and artificial intelligence. Recent studies have highlighted that multimodal approaches combining physiological signals such as Electrocardiography (ECG) and Photoplethysmography (PPG) contribute to improved cardiac output estimation and the prediction of hemodynamic instability [21]. Furthermore, developments in non-invasive cardiac output monitoring have demonstrated the potential for safer and more accessible hemodynamic assessment across a wide range of clinical settings, including intensive care units [22]. Despite these advancements, several limitations remain, including reduced accuracy under unstable hemodynamic conditions and variability associated with patient-specific factors and measurement principles [23]. Therefore, ongoing research continues to focus on improving the reliability, usability, and clinical applicability of non-invasive monitoring systems.

To address these limitations, carotid artery Doppler ultrasound (CDU) has emerged as a promising non-invasive hemodynamic monitoring modality [24,25]. The carotid artery serves as a major conduit for cerebral blood flow and reflects central hemodynamic status while being easily accessible due to its superficial anatomical location [24]. Previous studies have demonstrated that CDU examinations can be performed reliably even by novice ultrasound operators [24,26,27]. Nevertheless, conventional CDU techniques require continuous manual stabilization of the probe, making prolonged monitoring challenging. In addition, measurement variability related to the Doppler insonation angle introduces substantial inter-operator variability [24,28]. A comparative summary of the characteristics of these hemodynamic monitoring modalities is presented in Table 1.

**Figure 1:**
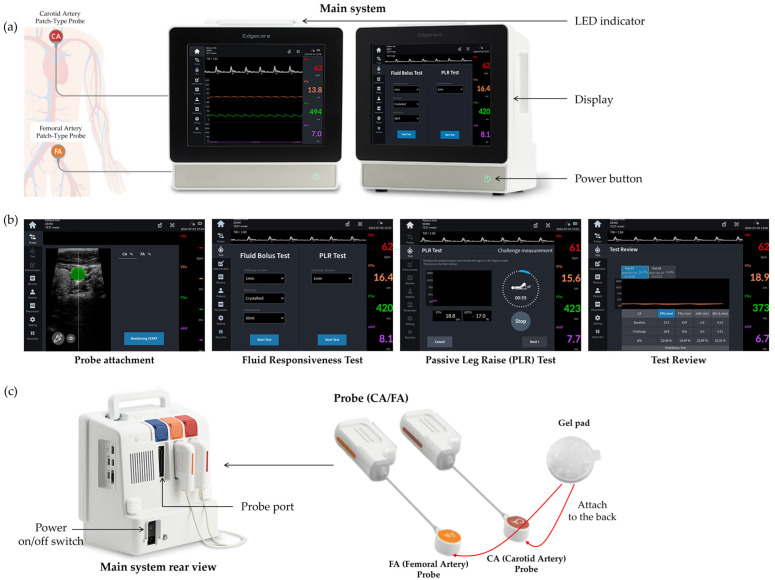
Real-time non-invasive patch-type ultrasound hemodynamic monitoring system (CW10): (**a**) Main system; (**b**) main screen; (**c**) probes attached to the rear of the main system and disposable gel pads.

Accordingly, there is a growing need for a novel approach that enables continuous and stable hemodynamic assessment while minimizing operator dependency. The patch-type ultrasound cardiac output monitoring system evaluated in this study (EdgeFlow CW10, Edgecare, Seoul, Republic of Korea) is a non-invasive monitoring device designed to address the technical limitations of conventional hand-held ultrasound systems, as illustrated in Figure 1. The system consists of a main system, ultrasound probes, and a gel pad. The main system incorporates software for ultrasound image acquisition and signal processing, through which hemodynamic parameters are calculated from the acquired ultrasound data and displayed on the monitor in real time. The probe transmits ultrasound energy to the target area and receives reflected signals, allowing visualization of internal vascular structures using Doppler spectra and B-mode imaging. To assist users in identifying the correct anatomical position of the common carotid artery, the system provides visual feedback by displaying the vessel cross-sectional alignment in green when the probe head achieves optimal alignment with the artery and in red when alignment is inadequate. For hemodynamic monitoring, two types of probes are available: a carotid artery (CA) probe and a femoral artery (FA) probe. After confirming vessel location using B-mode imaging, the probe head can be affixed to the skin, enabling continuous measurement of blood flow velocity in the target vessel using Doppler ultrasound technology. The gel pad is a non-sterile, adhesive solid medical gel that eliminates the air interface between the probe head and the skin, thereby enhancing ultrasound energy transmission.

EdgeFlow CW10 is designed to extend the clinical applicability of conventional carotid Doppler ultrasound (CDU) through several key technical features. First, the system employs a patch-type probe that is directly affixed to the skin, enabling continuous and hands-free hemodynamic monitoring. This design reduces operator workload while enhancing patient safety and comfort across a wide range of clinical settings. Second, real-time image analysis is performed using a cross-array ultrasound patch attached to either the carotid or femoral artery. When the user positions the target vessel at the center of the real-time B-mode cross-sectional image, an automated image analysis algorithm identifies the precise vessel location. Subsequently, two phased-array transducer modules acquire Doppler signals, and vector Doppler technology is applied to minimize the influence of insonation angle, thereby generating a stable Doppler spectrum. Third, through this signal processing workflow, the system derives heart rate (HR) and velocity–time integral (VTI), and continuously estimates cardiac output based on the vessel cross-sectional area (CSA) calculated from the B-mode cross-sectional image. These automated measurement and correction mechanisms are intended to mitigate variability associated with operator experience and to provide a technical foundation for supporting fluid management decision-making in hemodynamically unstable patients during surgical procedures.

### 1.2. Usability

According to the international standard IEC 62366-1:2015, medical device usability is defined as the characteristic of a user interface that enables intended users to achieve effectiveness, efficiency, and user satisfaction within the intended use environment [29]. Beyond mere convenience, usability constitutes a core component of risk management under ISO 14971, as it focuses on the identification and control of risks associated with use errors [30]. In medical devices that require rapid interpretation of information and immediate clinical decision-making, such as hemodynamic monitoring systems, the lack of a structured usability engineering process may be associated with user interface–related operational errors, which in turn may pose risks to patient safety.

Accordingly, this study conducted a summative usability evaluation as a key activity in the early stage of the medical device development lifecycle to identify potential use-related risks prior to clinical application and to inform design improvements.

## 2. Materials and Methods

### 2.1. Ethical Considerations

This study was conducted following approval from the Institutional Review Board (IRB) of Gangnam Severance Hospital, Yonsei University Health System (No. 3-2024-0347). Prior to participation, all participants were informed of the study objectives, procedures, and measures for the protection of personal information, and written informed consent was obtained voluntarily. All study procedures were carried out in accordance with relevant ethical standards and applicable guidelines.

### 2.2. Participants

The intended users of the system in this study were defined as healthcare professionals responsible for operating a real-time, non-invasive, patch-type ultrasound hemodynamic monitoring system. Accordingly, board-certified anesthesiologists were recruited as study participants. Participants were enrolled through expert referral using a snowball sampling approach and were required to be adults aged 19 years or older with prior experience in using hemodynamic monitoring systems [31]. To reflect diverse clinical environments and institutional practices, participants were recruited from eight different medical institutions rather than a single center.

The sample size was determined in accordance with the international standard IEC TR 62366-2:2016. According to this guideline, a cumulative probability (R) of approximately 0.91 (91%) for identifying at least one usability problem can be achieved with a sample size of 15 participants (*n* = 15). Consequently, a total of 15 healthcare professionals participated in the usability evaluation.

### 2.3. Study Design

#### 2.3.1. Summative Evaluation

This study was designed as a summative usability evaluation in accordance with the international standard IEC 62366-1:2015 and the U.S. Food and Drug Administration (FDA) guidance document Applying Human Factors and Usability Engineering to Medical Devices (2016) [29,32]. The primary objective of this evaluation was to verify that the user interface, refined through prior formative evaluations, operates safely and effectively, and to confirm that potential use errors associated with hazard-related use scenarios have been reduced to an acceptable level. The evaluation was conducted in a simulated use environment, and evidence was collected through usability testing involving observation and analysis of participants’ performance on predefined tasks.

#### 2.3.2. Use Scenario

To identify known or foreseeable hazards and hazardous situations associated with medical device use, post-market surveillance (PMS) data were analyzed using the FDA Manufacturer and User Facility Device Experience (MAUDE) database [33,34]. The analysis focused on reports corresponding to devices classified under the same FDA product code as the study device, namely DQK, defined as computer, diagnostic, programmable. Cases reported between 2021 and 2023 were reviewed and incorporated into the development of use scenarios (Table 2).

Based on the identified hazards, use scenarios reflecting actual clinical workflows were derived, comprising nine stages from (1) device preparation to (9) patient discharge, with a total of 49 detailed tasks (Table 3). All hazard-related scenarios were included in the evaluation. In addition, critical tasks were identified and analyzed with particular emphasis. According to the FDA Human Factors Engineering Guidance (2016), critical tasks are defined as user tasks that, if performed incorrectly or not performed at all, could result in serious harm to the patient or the user [32]. In this study, tasks with a hazard severity rating of level 3 or higher, as defined by the severity scale in ISO/TR 24971:2020 (Clause 5.5.5), were classified as critical tasks and included in the analysis [35].

#### 2.3.3. Test Environment

**Figure 2:**
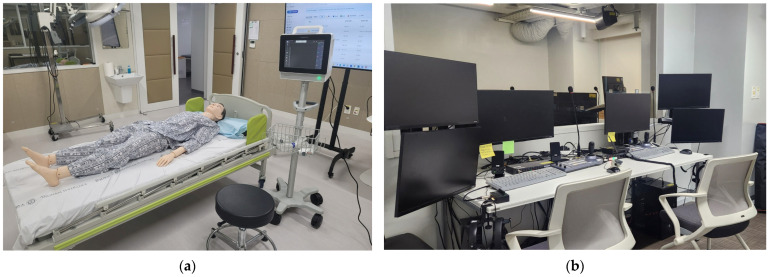
Configuration of the usability test environment: (**a**) Test room designed to simulate an actual operating room setting; (**b**) Observation room equipped for real-time monitoring and data recording.

The evaluation was conducted in a dedicated assessment space designed to simulate an actual operating room environment (Figure 2).

The test setting included a patient bed, a patient mannequin, and the study device to ensure physical similarity to real clinical conditions. To standardize environmental conditions, the room temperature was maintained between 18 and 23 °C and relative humidity between 30 and 60%, reflecting typical operating room standards [36,37]. The evaluation area and observation area were separated by a one-way mirror to minimize external interference with participant behavior. The entire usability evaluation process was recorded in both video and audio formats using Media Express software (version 1.0, Blackmagic Design, Melbourne, Australia).

#### 2.3.4. Procedure

The usability evaluation was conducted in individual one-on-one sessions for each participant. One facilitator and two evaluators (an observer and a data analyst) were involved in the assessment. The observer and data analyst monitored the entire session from the observation room and systematically documented relevant information using structured observation sheets. The collected observational data were subsequently used for post hoc analysis.

The evaluation procedure began with an explanation of the study objectives and assessment process, followed by the acquisition of written informed consent. Participants then completed a pre-test questionnaire to collect demographic information. Subsequently, participants received training on the key functions and basic operation of the study device. The training session lasted approximately 15 min and was conducted following a standardized procedure, including probe attachment, screen layout, and basic operational functions.

Following the training, participants were given a 5 min familiarization period to freely interact with the device. Repeated practice of predefined evaluation scenarios and specific tasks was intentionally restricted.

To minimize the influence of training on evaluation outcomes, a time interval was introduced after the training session in accordance with the recommendations of Annex K of IEC 62366-1. Specifically, a 10 min decay period was implemented to mitigate short-term memory effects immediately following training and to reflect a learning decay condition comparable to real-world use environments [29].

After the waiting period, participants sequentially performed nine predefined use scenarios comprising a total of 49 detailed tasks. Upon completion of all scenarios, task-specific subjective satisfaction ratings and the System Usability Scale (SUS) questionnaire were administered. This was followed by in-depth interviews to identify and analyze the root causes of observed use errors and usability-related difficulties.

### 2.4. Data Collection and Analysis

#### 2.4.1. Task Success Rate

In this study, task performance outcomes were classified into three categories for the analysis of task success rate: Complete (C), Complete with Issues (CI), and Not Complete (NC). This classification scheme represents an operational definition established for the systematic organization of task performance results in the present study. As shown in Equation (1), the task success rate was calculated as the proportion of successfully completed tasks (C and CI) relative to the total number of task attempts (P*_n_*).

(1) $$Task\;Success\;Rate\left(\%\right)=\frac{C+CI}{P_{n}}\times 100,$$

A task was classified as Complete (C) when the task objective was achieved without any observable usability-related issues. Complete with Issues (CI) was defined as cases in which the final task goal was achieved, but close calls or user difficulties were observed during task execution. In contrast, Not Complete (NC) referred to tasks in which the intended goal was not achieved, including instances in which participants requested assistance from the facilitator or when a use error occurred. The interpretation of close calls, user difficulties, and use errors was based on established usability engineering and human factors guidance documents [29,32,38].

#### 2.4.2. Ease of Use and Satisfaction

A subjective usability questionnaire was developed to evaluate whether the device’s user interface functions effectively within actual clinical settings.

The questionnaire was grounded in the three core components of usability defined by ISO 9241-210: effectiveness, efficiency, and satisfaction [37]. To tailor the evaluation framework for medical device interfaces, these core concepts were integrated with Nielsen’s ten usability heuristics and the Goal–Question–Metric (GQM) framework, resulting in the derivation of five specific measurement metrics [39,40].

The questionnaire was administered upon the completion of all tasks within the predefined use scenarios. Participants were asked to rate their level of agreement based on their overall task performance experience. The specific measurement intent and theoretical mapping for each metric are summarized in Table 4. All items were assessed using a five-point Likert scale, with higher scores indicating greater satisfaction. In the data analysis, mean values along with minimum and maximum scores were calculated. Items receiving low ratings were further examined through follow-up interviews to identify the root causes of errors.

#### 2.4.3. System Usability Scale

The System Usability Scale (SUS) was administered to quantitatively assess the overall usability of the device. As a standardized ten-item instrument, this scale is widely utilized in usability research for the rapid and efficient evaluation of various products and services. The scale features alternating positively and negatively worded statements, each rated on a five-point Likert scale [41]. Responses were converted into a standardized score ranging from zero to 100 according to the established scoring procedure.

Specifically, for positively worded items, one was subtracted from the user’s response, whereas for negatively worded items, the response value was subtracted from five. The sum of these adjusted scores was then multiplied by 2.5 to determine the final score. The resulting scores were interpreted based on the adjective ratings and acceptability ranges proposed by Bangor. Generally, a score of 68 is considered the minimum threshold for acceptable usability, while a score of 85 or higher indicates an excellent level of usability [41,42].

### 2.5. Statistical Analysis

Statistical analyses were conducted using IBM SPSS Statistics (version 31.0, IBM Corp., Armonk, NY, USA). Descriptive statistics, including means and standard deviations, were calculated for all evaluation metrics. This study was designed as a summative usability evaluation involving 15 participants, and the primary analyses were performed with all participants treated as a single cohort.

To examine potential differences based on clinical experience with hemodynamic monitoring systems, participants were stratified into three groups of 5 according to years of experience. Group 1 consisted of participants with 1 to 5 years of experience, Group 2 included those with 5 to 10 years of experience, and Group 3 comprised participants with 10 or more years of experience.

For between-group comparisons, Fisher’s exact test was applied to task success rates, while the Kruskal–Wallis test was used to analyze ease-of-use scores, satisfaction ratings, and System Usability Scale scores. When statistically significant differences were identified, post hoc multiple comparisons with Bonferroni correction were performed. Statistical significance was defined as *p* < 0.05.

## 3. Results

### 3.1. User Statistics

A total of 15 board-certified anesthesiologists participated in the evaluation, representing the intended user group for the real-time non-invasive patch-type hemodynamic monitoring system. All participants were practicing physicians employed at medical institutions in the Republic of Korea, and their detailed demographic characteristics are summarized in Table 5. All participants possessed more than 3 years of experience in operating ultrasound-based devices, indicating a sufficient baseline level of proficiency for device operation. To examine the potential influence of professional experience with comparable technologies, participants were evenly allocated into three groups of 5 based on their prior experience with similar hemodynamic monitoring systems, including systems manufactured by Edwards Lifesciences and Masimo. This classification served as a key independent variable for subsequent between-group comparisons.

### 3.2. Task Performance and Success Rate

#### 3.2.1. Overall Success Rate

The 15 anesthesiologists performed 49 detailed tasks across 9 predefined use scenario stages. The overall mean task success rate was 98.2%. Detailed task success and failure rates for each scenario stage are presented in Table 6. Scenario-specific success rates ranged from 94.8% to 100%, with the Monitor the Patient stage demonstrating the lowest success rate at 94.8%.

Among the 49 subtasks, failures or requests for facilitator assistance were observed in 7 instances, while 10 tasks were completed with difficulties classified as complete with issues. Several participants reported a need for improved visual indicators to better distinguish signal strength levels. Although the patch-type probe-based monitoring approach was positively evaluated in terms of operational convenience, certain usability challenges were observed during the gel pad attachment process.

#### 3.2.2. Root Cause Analysis

A total of 13 task failures classified as NC and 17 tasks classified as CI were identified. These cases were categorized by critical task status and error type, and the root causes of errors were analyzed accordingly. The classification results are summarized in Table 7, and the detailed distribution of close calls and user difficulties is presented in Table 8.

The analysis showed that several use errors were associated with negative transfer, in which operational habits formed through prior experience with conventional monitoring devices were applied to the CW10 interface. For example, in Tasks 1.4 and 1.5, several participants connected the probes incorrectly, reflecting prior experience with other devices in which the probe cable was oriented upward during connection. Similarly, unnecessary navigation into menu paths, including the Parameter Setting menu, was observed when users followed interaction patterns familiar from legacy systems.

In addition to negative transfer, Table 8 indicates that limited visual salience of certain interface elements and non-intuitive interaction designs were associated with user difficulties. Observed issues included delays in closing the digital keypad related to the use of the Enter and Hide buttons in Task 3.1, difficulty locating the Scan button in Task 5.3, and attempts to select non-interactive records in Tasks 8.3 and 8.4.

Use errors occurring during critical tasks, including improper probe alignment and failure to maintain the Signal Strength Indicator, were observed to have the potential to delay monitoring initiation. However, no instances of complete monitoring interruption were recorded during the evaluation.

### 3.3. Statistical Analysis by Clinical Experience

#### 3.3.1. Comparison of Task Success Rate

To examine differences in task performance based on the duration of experience with hemodynamic monitoring systems, Fisher’s exact test was applied to the 7 subtasks in which NC outcomes were observed. The analysis showed no statistically significant differences in success rates between groups (*p* > 0.05).

Specifically, for all evaluated items, including the critical tasks related to probe connection (Tasks 1.4 and 1.5) and maintenance of the signal strength indicator (Task 5.4), *p*-values ranged from 0.725 to 1.000. These results indicate that no statistically significant differences in task success rates were observed between groups within the sample of this study. Detailed group-level results are presented in Table 9.

#### 3.3.2. Comparison of Ease of Use and Satisfaction

Subjective evaluations conducted immediately after scenario completion revealed no statistically significant differences between groups (*p* > 0.05). When the five evaluation dimensions—intuitiveness, effectiveness, visibility, simplicity, and overall satisfaction—were analyzed collectively, users across all experience levels perceived the interface elements to offer a comparable level of usability.

Given the small sample size of five participants per group, the Kruskal–Wallis test was applied. The resulting *p*-values across all scenarios ranged from 0.362 to 0.980, as detailed in Table 10.

#### 3.3.3. Comparison of SUS Scores

The mean SUS score across all participants was 78.5 (SD = 14.54). According to the adjective rating scale proposed by Bangor and colleagues, this score corresponds to good usability and falls within the acceptable range.

When scores were compared according to experience level, Group 1 demonstrated a mean score of 80.5 (SD = 13.85), Group 2 showed a mean score of 74.5 (SD = 11.65), and Group 3 showed a mean score of 80.5 (SD = 20.87). The Kruskal–Wallis test revealed no statistically significant differences between groups (*p* = 0.664). Analysis of the 10 individual items also revealed no statistically significant differences, with all *p*-values exceeding 0.05. Detailed group-level comparisons are presented in Table 11.

## 4. Discussion

This study was conducted as a summative usability evaluation applying established testing methods to verify whether the user interface of a patch-type non-invasive hemodynamic monitoring system can be used safely and effectively by its intended users. The results demonstrated a high overall mean task success rate of 98.2 percent, and the mean SUS score was 78.5, corresponding to a good usability rating. These findings suggest that the user-centered design approach and iterative refinement process recommended by IEC 62366-1 may translate into stable task performance even under simulated clinical use conditions [29].

The high task success rate observed in this summative evaluation indicates that user interactions with the device were performed consistently and successfully across the predefined use scenarios. Consistent execution was observed during procedures such as patient registration, ultrasound scanning, fluid responsiveness assessment, and data review, suggesting that the system may provide a level of operational stability under simulated clinical conditions. In particular, the stages related to “Progressing the Scan” (98.3%) and “Monitoring the Patient” (94.8%) represent key workflow components that distinguish this system from conventional hand-held ultrasound approaches. These stages are especially relevant as they reflect the conditions of continuous hemodynamic monitoring. Although close calls and user difficulties were observed in certain tasks, these issues were not observed to adversely affect overall system operation during the evaluation and may serve as informative inputs for future design refinements.

No statistically significant differences were observed across user groups stratified by years of experience with hemodynamic monitoring devices in terms of task success rates, subjective ease-of-use ratings, or SUS scores. Between-group comparisons were conducted as exploratory analyses, and an a priori power analysis was not included in the study design. In addition, the analysis was limited to tasks in which NC outcomes were observed. Conventional hemodynamic monitoring techniques, such as pulmonary artery catheterization and manual Doppler-based measurements, are known to be highly operator-dependent, with considerable variability in data acquisition and interpretation depending on users’ experience levels [43]. These findings suggest that the automated vessel localization algorithm, vector Doppler technology, and automated correction mechanisms implemented in this system may contribute to reducing inter-user variability. These findings are consistent with previous studies reporting that point-of-care ultrasound (POCUS) systems incorporating artificial intelligence and automation can improve operational consistency and accuracy among less experienced users [43]. A similar pattern was observed in ease-of-use assessments, where the ultrasound scanning item received consistently high ratings across all groups (M = 4.36, SD = 1.06 for Group 1; M = 4.41, SD = 0.79 for Group 2; M = 4.27, SD = 1.14 for Group 3).

These findings may be partly attributable to the system’s design. Unlike conventional hand-held ultrasound devices, the adhesive patch-type probe eliminates the need for continuous manual handling. Similarly, in a study by Kenny et al. (2021) evaluating the usability of a carotid-attached wearable Doppler patch, all participants, including both clinical experts and lay users, were able to easily acquire Doppler signals without the need for angle adjustment and reported high ease of use [44].

In the present study, user feedback indicated that monitoring ultrasound images while the probe remained attached was perceived as convenient, suggesting that the hands-free operation may offer potential benefits in reducing both physical and operational workload for users.

The SUS score obtained in this study (78.5) exceeds the commonly referenced acceptability threshold of 68 in medical device usability evaluations and corresponds to a “Good” level of usability [41]. For example, Barbosa et al. (2023) reported a SUS score of 86.5 in a study evaluating the usability of a smartphone application designed to support hemodynamic education and pulse pressure variation (PPV) measurement [45]. Although the SUS score of the present system is somewhat lower than that of purely software-based applications, the system involves direct probe attachment and real-time data acquisition.

Root cause analysis further revealed that some use errors were associated with negative transfer arising from prior experience with other commercial devices. For example, navigating unnecessary menu paths occurred when users applied familiar patterns from legacy systems to the new interface. This suggests that certain errors stem not from a lack of proficiency, but from mismatches between existing mental models and the new system’s design.

Taken together, the findings of this study suggest that the system suggests a level of interface usability for intended users, while potentially reducing cognitive and operational workload. In particular, the operational efficiency provided by the hands-free approach was identified as a favorable usability characteristic. These results provide preliminary usability-based insights, and the applicability of this approach in environments requiring continuous monitoring should be further investigated through future clinical validation studies.

In this usability evaluation, anesthesiologists were recruited from eight different hospitals. This recruitment strategy partially strengthens the external validity of the findings by incorporating diverse clinical backgrounds. However, several limitations should be considered. First, due to the nature of snowball sampling, the sample may have been biased toward specific networks, and sampling bias cannot be entirely excluded. In addition, the limited sample size may restrict the generalizability of the findings. Second, all evaluations were conducted within a single physical and organizational setting. As a result, institution-specific infrastructure, workflows, and team interactions in real clinical environments may not have been fully reflected, which may limit the ecological validity of this study. Third, all participants were anesthesiologists from a single country, which may not fully capture variations in clinical practices across countries or the characteristics of other user groups, such as intensivists and nurses.

Future studies should adopt multi-center study designs that include evaluations conducted in real clinical environments and involve a broader range of healthcare professionals beyond anesthesiologists, such as nurses and physicians from various specialties, to strengthen the evidence regarding the system’s usability and safety across diverse clinical contexts.

## 5. Conclusions

This study was conducted to evaluate the usability of a patch-type ultrasound–based hemodynamic monitoring system. The results demonstrated a high task success rate and favorable usability scores, with no statistically significant differences observed according to users’ experience levels. These findings suggest that the system may be used in a consistent manner by its intended users and indicate that the automated functions and adhesive patch–based probe design may contribute to reducing variability in device operation. In particular, the hands-free approach shows potential for application in clinical environments where continuous hemodynamic monitoring is required. Further studies incorporating real-world clinical settings are warranted to more comprehensively assess the system’s applicability across diverse users and use conditions. In this context, the user-centered evaluation approach applied in this study may provide a useful foundation for establishing usability-based assessment strategies in the development and validation of future medical devices.

## Figures and Tables

**Table 1:** Comparison of invasive, minimally invasive, and non-invasive hemodynamic monitoring technologies.

Category	Technology	Clinical Significance	Advantages	Limitation
Invasive	Pulmonary Artery Catheter (PAC)	Clinical gold standard for Cardiac Output (CO) measurement	High reliability even in unstable hemodynamic states such as shock	Risk of complications due to catheter insertion (e.g., infection, arrhythmia, pulmonary artery rupture)
	Transpulmonary Thermodilution	CO measurement via thermodilution and pulse wave analysis calibration	Less invasive than PAC Useful for evaluating pulmonary edema and fluid overload states	Frequent recalibration required when patient conditions change (e.g., vascular tone, body temperature
Minimally invasive	Uncalibrated Pulse Wave Analysis	CO estimation by analyzing the area and shape of peripheral arterial waveforms Utilizes algorithms based on patient characteristics without external calibration	Eliminates the need for a central venous catheter; can utilize existing arterial lines [5] Easy to use and less invasive	Reduced accuracy in conditions with rapid changes in vascular resistance or compliance (e.g., use of vasopressors, sepsis)
	Esophageal Doppler	Evaluation of descending aortic blood flow via an esophageal probe	Assessment of fluid responsiveness and detection of rapid blood flow changes	Visualization of cardiac structure, contractility, and valvular function
	Transesophageal Echocardiography (TEE)	Evaluation of cardiac structure and function via an esophageal probe	Direct visualization of cardiac anatomy, contractility, and valvular function	Difficulty in maintaining probe position and high operator dependency
Non-invasive	Transthoracic Echocardiography (TTE)	Non-invasive visualization of cardiac structure and function	Safe and provides intuitive imaging information	Requires manual operation by an expert; continuous monitoring is impossible Image quality is highly operator-dependent
	Volume Clamp	Reconstruction of blood pressure waveforms and CO measurement via finger arterial pressure	Enables continuous monitoring without catheterization No risk of infection	Signal acquisition failure and reduced accuracy in patients with peripheral vasoconstriction (e.g., shock, hypothermia) or edema
	Bioimpedance/Bioreactance	Measurement of electrical resistance/phase changes based on blood flow variations via thoracic electrodes	Simple measurement through electrode attachment alone High safety profile	Vulnerable to electrical noise, patient movement, and pulmonary edema; accuracy remains controversial
	Carotid Artery Doppler Ultrasound (CDU)	Evaluation of blood flow (velocity time integral, VTI) in the carotid artery to assess CO and fluid responsiveness	Non-invasive relatively easy for beginners to perform	Requires manual operation; continuous monitoring is impossible Measurement errors due to the Doppler angle of incidence

**Table 2:** Analysis of FDA MAUDE data for product code DQK to identify potential hazards.

Classification	No.	Description of Event
Cable & Cord	1	Intermittent disconnection occurred; replacing the ECG cable resolved the issue.
	2	The display monitor cable was loosely connected.
	3	The cable was damaged during the procedure after becoming entangled with a staff member’s foot.
Patch	4	Due to excessive adhesion, a minor skin tear occurred upon patch removal.
	5	A connector pin in the patch module was bent.
	6	Skin tearing and bruising occurred at the ground pad site during removal.
	7	Map shift occurred due to the displacement of the anterior patches.
User error	8	The HemoSphere monitor was inadvertently set to “Demo Mode”, and the on-screen notification went unnoticed. Patient care proceeded under simulation settings.
	9	An incorrect catheter size was used.
Display	10	The device failed to display errors or alerts despite significant map shifts.
	11	Continuous Cardiac Output (CCO) could not be measured, and no error messages were displayed.
Overheat	12	Outlet overheating occurred due to user error (power overload); a high-power device was connected incorrectly.
Sensor	13	A sensor was found to be loose.
Software	14	System failure occurred due to a missing software update.

**Table 3:** Use scenarios and subtasks for the summative usability evaluation.

Use Scenarios	No.	Task
Preparation for Use	Task 1.1 *	Locate the “Caution” section in the Safety Information of the user manual.
	Task 1.2	Locate the “Main System” section in the user manual.
	Task 1.3	Locate the “Instructions for Use” section in the user manual.
	Task 1.4 *	Connect CA probe to Main system.
	Task 1.5 *	Connect FA probe to Main system
	Task 1.6	Connect the power cord to the main system, verify that it is properly connected, and position the cables appropriately.
Turn On the Power	Task 2.1	Turn on the power switch and press the power button.
Register The Patient	Task 3.1	Register a new patient.
Modifying The Patient Information	Task 4.1	Modify the patient ID.
Progress The Scan *	Task 5.1	Open the Probe menu.
	Task 5.2	After applying an appropriate amount of ultrasound gel to the CA probe head, align the marking guide (Carotid Artery Alignment Line) printed on the top surface of the CA probe head parallel to the carotid artery.
	Task 5.3	Press the Scan button.
	Task 5.4	Place the probe to an appropriate position utilizing the video. The mark on the screen will turn green if the vertical line comes to the center of vessel cross-section and the SSI exceeds 0.5, the indicator will turn green.
	Task 5.5	Attach the guideline sticker align to the attach-assisting line of the probe.
	Task 5.6	Remove the gel from the patient’s region and the probe.
	Task 5.7	Unpack the gel pad and remove the release film.
	Task 5.8	Attach the gel pad to the probe head, attach it evenly without air bubble.
	Task 5.9	Remove the release film of the other side of the gel pad.
	Task 5.10	Attach the probe by aligning the attach-assisting line and the guideline sticker.
	Task 5.11	Start monitoring.
	Task 5.12	Check the Doppler spectrum to decide if probe repositioning is necessary.
Monitor The Patient	Task 6.1	During the ultrasound scanning process, temporarily lock the main screen to wipe off fingerprints.
	Task 6.2	Unlock the main screen.
	Task 6.3	Change the parameter color of HRc.
	Task 6.4	Change the parameter (HRf, VTIc → HRc, VTIf) of Main screen.
	Task 6.5	Change the parameter layout to 6 parameter screens.
	Task 6.6	Capture the snapshot.
	Task 6.7	Touch the waveform to view the parameter values.
	Task 6.8	Remove the appeared parameter value.
	Task 6.9	Adjust the screen brightness.
Conduct One of The Fluid Responsiveness Test; Fluid Bolus Test or PLR Test *	Task 7.1	Conduct the Fluid Bolus Test as follows: - Challenge duration: 1 min - Type of fluid: Crystalloid - Volume of fluid: 100 mL Conduct the PLR Test as follows - Challenge duration: 1 min
	Task 7.2	Measure the baseline. Or, Using the bed, raise the upper position of the body for 45 degree, and measure the baseline.
	Task 7.3	You have noticed the problem of the baseline measurement. Restart the baseline measurement.
	Task 7.4	If the measurement is sufficient, proceed to the next step.
	Task 7.5	Start Fluid Challenge. Or, Lower the upper body and elevate the lower body to a 45 degree angle using the bed to begin the PLR Test.
	Task 7.6	If the measurement is sufficient, check the result.
Review The Monitoring	Task 8.1	Check the record of PLR or Fluid Bolus in Event Section.
	Task 8.2	(Assume using vasopressor) Record the Intervention.
	Task 8.3	Check if the intervention has recorded correctly in the Review screen.
	Task 8.4	Use the copy function to add a new record and enter the initials as a memo.
	Task 8.5	Check the captured screen.
	Task 8.6	Before exporting the monitoring records so far, you want to review the previous values. Check the review screen
	Task 8.7	Insert the USB into the Main system.
	Task 8.8	Press the export button.
	Task 8.9	Enter the password; 1234.
	Task 8.10	Select the inserted USB from the drop box and press export button with the content.
Discharge The Patient	Task 9.1 *	Remove the probe from the patient.
	Task 9.2	Discharge the patient.
	Task 9.3	Shutdown the power.

* Asterisks (*) indicate critical tasks, which are defined as tasks where use errors could potentially lead to significant harm or medical risks to the patient or user.

**Table 4:** Derivation of usability evaluation metrics and their theoretical mapping.

Item	Survey Statement (Presented to Participants)	Theoretical Basis
Intuitiveness	The UI structure is easy to understand and use without significant effort.	Nielsen (Consistency)
Effectiveness	Users’ goals can be achieved accurately and completely.	ISO 9241-210
Visibility	Key functions are clearly displayed to make operations as easy as possible.	Nielsen (Visibility)
Simplicity	The functional structure is simplified to minimize operational effort and reduce cognitive load.	GQM (Simplicity)
Satisfaction	Overall satisfaction regarding the use of the device’s functions.	ISO 9241-210

**Table 5:** Demographic and professional characteristics of the participants (*n* = 15).

Variable	Category	*n* (%)
Age	30–39 years	11 (73.3)
	40–49 years	4 (26.7)
Sex	Male	8 (53.3)
	Female	7 (46.7)
Total clinical experience	1 to <5 years	5 (33.3)
	5 to <10 years	4 (26.7)
	≥10 years	6 (40.0)
Experience with similar Devices *	1 to <5 years	5 (33.3)
	5 to <10 years	5 (33.3)
	≥10 years	5 (33.3)
Experience with ultrasound	3 to <10 years	8 (53.3)
	≥10 years	7 (46.7)
Echocardiography experience **	TEE	12 (80.0)
	TTE	5 (33.3)
Experience with Carotid Ultrasound	Yes	11 (73.3)
	No	4 (26.7)

* Participants were grouped based on their experience with similar hemodynamic monitoring systems (e.g., Edwards Lifesciences, Masimo) for statistical analysis. ** Percentages may exceed 100% due to multiple responses.

**Table 6:** Task completion rate by scenario (*n* = 15).

Use Scenarios	No. of Tasks	Task Pass Rate (%)	Task Failure Rate (%)
Preparation for Use	6	97.7%	2.3%
Turn On the Power	1	100.0%	0.0%
Register The Patient	1	100.0%	0.0%
Modifying The Patient Information	1	100.0%	0.0%
Progress The Scan	12	98.3%	1.7%
Monitor The Patient	9	94.8%	5.2%
The Fluid Responsiveness Test	6	100.0%	0.0%
Review The Monitoring	10	99.3%	0.7%
Discharge The Patient	3	100.0%	0.0%
Overall rate	49	98.2%	1.8%

**Table 7:** Summary of use errors and root cause analysis.

Type	Tasks No.	Use Error	Root Cause
NC (Critical Tasks)	1.4, 1.5	Probe connected upside down	Negative transfer from prior experience with other devices
	5.4	Failed to maintain SSI * above 0.5	Lack of familiarity with the new device interface
	5.7	Gel pad crumpled during film removal	Confusion regarding the multi-layered film structure
NC (Non-critical Tasks)	6.4	Entered ‘Parameter Setting’ unnecessarily	Habitual reliance on conventional UI structures
	6.7	Clicked Doppler spectrum instead of waveform	Visual priority given to faster-loading Doppler signals
	8.4	Failed to locate ‘Current Entries’	Ambiguity in menu navigation and layout

* The Signal Strength Indicator (SSI) refers to the numerical value representing the signal intensity of the Doppler measurement.

**Table 8:** Summary of close calls, difficulty and root cause analysis.

Type	Tasks No.	Close Call/Difficulty	Root Cause
CI	3.1	Delay in closing keypad	Non-intuitive ‘Enter’ and ‘Hide’ button design
	5.3 *	Difficulty locating the ‘Scan’ button	Low visual salience of the interface element
	7.4 *	Pressed ‘Next’ instead of ‘Stop’	User’s intent to proceed outweighed the UI logic
	8.3, 8.4	Attempted to click non-interactive records	Misperception of non-clickable areas as interactive

* Asterisks (*) denote critical tasks, where any performance failure could potentially lead to significant medical risk or patient harm.

**Table 9:** Comparison of success rates for tasks with observed use errors.

Task No.	Group 1, C/NC (%)	Group 2, C/NC (%)	Group 3, C/NC (%)	*p*-Value
1.4 *	5/0 (100%)	4/1 (80%)	5/0 (100%)	1.000
1.5 *	4/1 (80%)	4/1 (80%)	5/0 (100%)	1.000
5.4 *	4/1 (80%)	4/1 (80%)	5/0 (100%)	1.000
5.6	5/0 (100%)	5/0 (100%)	4/1 (80%)	1.000
6.4	5/0 (100%)	4/1 (80%)	3/2 (60%)	0.725
6.7	4/1 (80%)	3/2 (60%)	4/1 (80%)	1.000
8.4	4/1 (80%)	5/0 (100%)	5/0 (100%)	1.000

* Asterisks (*) denote critical tasks, where any performance failure could potentially lead to significant medical risk or patient harm.

**Table 10:** Comparison of ease of use and satisfaction scores by scenario stage.

No.	Evaluation Item	Group 1, Mean (±SD)	Group 2, Mean (±SD)	Group 3, Mean (±SD)	*p*-Value
1	Preparation for use	4.66 ± 0.75	4.61 ± 0.74	4.63 ± 0.64	0.561
2	Patient registration	4.76 ± 0.60	4.92 ± 0.28	4.88 ± 0.44	0.546
3	Ultrasound scan	4.36 ± 1.06	4.41 ± 0.79	4.27 ± 1.14	0.907
4	Hemodynamic monitoring	4.86 ± 0.47	4.85 ± 0.47	4.66 ± 0.59	0.882
5	Fluid responsiveness assessment	4.80 ± 0.58	4.96 ± 0.20	4.64 ± 0.76	0.980
6	Fluid Bolus Test or PLR Test	4.50 ± 1.07	5.00 ± 0.00	4.58 ± 0.78	0.362
7	Data review	4.52 ± 0.90	4.93 ± 0.28	4.66 ± 0.61	0.670
Overall score		4.64 ± 0.24	4.81 ± 0.29	4.62 ± 0.22	0.386

**Table 11:** Comparison of item-specific SUS scores by group.

No.	Usability Item	Group 1, Mean (±SD)	Group 2, Mean (±SD)	Group 3, Mean (±SD)	*p*-Value
SUS 1	I think that I would like to use this system frequently.	3.40 ± 0.55	3.00 ± 0.55	2.60 ± 1.52	0.651
SUS 2	I found the system unnecessarily complex (R) *.	2.80 ± 1.10	3.80 ± 1.22	3.40 ± 0.89	0.180
SUS 3	I thought the system was easy to use.	3.80 ± 0.45	3.40 ± 0.45	2.80 ± 1.30	0.282
SUS 4	I think that I would need the support of a technical person to be able to use this system (R) *.	2.00 ± 1.58	1.20 ± 1.30	3.20 ± 0.45	0.072
SUS 5	I found the various functions in this system were well integrated.	3.40 ± 0.55	3.40 ± 0.84	3.60 ± 0.55	0.856
SUS 6	I thought there was too much inconsistency in this system (R) *.	3.80 ± 0.45	4.00 ± 0.00	3.40 ± 1.34	0.581
SUS 7	I imagine that most people would learn to use this system very quickly.	3.60 ± 0.55	2.60 ± 1.30	3.20 ± 1.34	0.299
SUS 8	I found the system very cumbersome to use (R) *.	3.60 ± 0.55	2.60 ± 0.89	3.60 ± 1.30	0.335
SUS 9	I felt very confident using the system.	3.00 ± 0.71	2.60 ± 0.45	2.80 ± 1.10	0.857
SUS 10	I needed to learn a lot of things before I could get going with this system (R) *.	2.80 ± 1.30	3.20 ± 0.84	3.60 ± 0.55	0.533
Group SUS score on 0 to 100 normalized scale		80.50 ± 13.85	74.50 ± 11.65	80.50 ± 20.87	0.664
Overall SUS score on 0 to 100 normalized scale		78.50 ± 14.54			

* (R) indicates reverse-scored items. For these negative statements (even-numbered items), a lower raw score represents a more positive usability result.

## Data Availability

The data presented in this study are available on request from the corresponding author due to privacy and ethical restrictions, as they involve human participants.

## Abbreviations

The following abbreviations are used in this manuscript:

IEC	International Electrotechnical Commission
ISO	International Organization for Standardization
FDA	Food and Drug Administration
UI	User Interface
C	Complete
CI	Completed with Issues
NC	Not Complete
SUS	System Usability Scale
